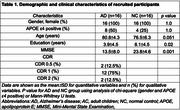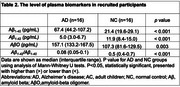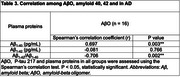# Using plasma amyloid oligomer to screen Alzheimer's disease

**DOI:** 10.1002/alz70856_099790

**Published:** 2025-12-24

**Authors:** Pin‐Chieh Hsu, Jia‐Ying Yang, Yuan‐Han Yang

**Affiliations:** ^1^ Kaohsiung Medical University, Kaohsiung, Kaohsiung, Taiwan; ^2^ Kaohsiung Medical university, Kaohsiung, Kaohsiung, Taiwan; ^3^ Kaohsiung Medical University Gangshan Hospital, Kaohsiung, Taiwan; ^4^ Neuroscience Research Center, Kaohsiung Medical University, Kaohsiung, TAIWAN, Taiwan; ^5^ Kaohsiung Medical University Hospital, Kaohsiung Medical University, Kaohsiung City, Taiwan; ^6^ Master's Program of Neurology, Faculty of Medicine, College of Medicine, Kaohsiung Medical University, Kaohsiung, Taiwan

## Abstract

**Background:**

Previous studies have shown that plasma amyloid‐beta oligomers(AβO), the toxic form of Aβ found in the blood, as a critical issue to develop or worsen Alzheimer's disease (AD). We have examined plasma amyloid‐beta oligomers and their related biomarkers in a case‐control study to clarify these issues.

**Method:**

A total of 16 patients with Alzheimer's disease (AD) dementia and 16 cognitively normal controls (NCs) were included to compare plasma biomarkers such as AβO, Aβ_1‐40_, and Aβ_1‐42_. The antibodies used for plasma biomarker detection were purchased from Shanghai Jinze Biotechnology Co., Ltd for oligomers analysis, and other ELISA kits (Human Amyloid β (1–40) Assay Kit–Invitrogen, code number KHB3481, Human Amyloid β (1–42) Assay Kit–Invitrogen, code number, KHB3441 were applied. Demographic and clinical variables, including age, biological sex, years of education, APOE ε4 status, clinical dementia rating (CDR), and mini‐mental status examination (MMSE) scores, were analyzed for both groups. Spearman's correlation test was applied to examine the relationships among AβO, and plasma proteins across all participants.

**Result:**

The pilot study recruited 16 AD patients with the disease severity of AD, CDR 0.5=2, CDR 1=12, CDR 2=2, compared to 16 normal healthy controls (NC). The CDR‐SB scores of the recruited participants were predominantly concentrated at 1, indicating that the majority of the subjects exhibited a mild degree of Alzheimer's dementia. The mean age of AD was 80.8±4.3 vs 76.5±6.3 in NC (*p* = 0.051). In plasma concentration of Aβ_1‐40_, Aβ_1‐42_, and Aβ oligomer, the mean concentrations were significantly different between the two groups (AD vs NC), Aβ_1‐40_: 67.4 pg/mL vs 21.4 pg/mL (*p* <0.001); Aβ_1‐42_: 5 pg/mL vs 11.9 pg/mL (*p* <0.0001), and AβO: 157.1 pg/mL vs 107.3 pg/mL (*p* = 0.003). The Spearman's correlation coefficients for Aβ_1‐40_, Aβ_1‐42_, and the Ab1‐42/1‐40 ratio to AβO were 0.697 (*p* = 0.003), ‐0.081(*p* = 0.766), and ‐0.706 (*p* = 0.002), respectively. However, the *p*‐value for Aβ_1‐42_in the Spearman's correlation (*p* < 0.05) was 0.766.

**Conclusion:**

Higher plasma concentrations of Aβ oligomers were significantly associated with Alzheimer's disease (AD) compared to non‐demented controls. This suggests that Aβ oligomers(AβO) can be potential plasma biomarkers to screen AD. However, a study recruiting more individuals is necessary to examine the association.